# When Does Hardship Matter for Health? Neighborhood and Individual Disadvantages and Functional Somatic Symptoms from Adolescence to Mid-Life in the Northern Swedish Cohort

**DOI:** 10.1371/journal.pone.0099558

**Published:** 2014-06-12

**Authors:** Per E. Gustafsson, Miguel San Sebastian

**Affiliations:** 1 Department of Public Health and Clinical Medicine, Family Medicine, Umeå University, Umeå, Sweden; 2 Department of Public Health and Clinical Medicine, Epidemiology and Global Health, Umeå University, Umeå, Sweden; Tilburg University, Netherlands

## Abstract

A large body of research has shown that health is influenced by disadvantaged living conditions, including both personal and neighborhood conditions. Little is however known to what degree the health impact of different forms of disadvantage differ along the life course. The present study aims to examine when, during the life course, neighborhood and individual disadvantages relate to functional somatic symptoms. Participants (n = 992) came from The Northern Swedish Cohort and followed from age 16, 21, 30 until 42 years. Functional somatic symptoms, socioeconomic disadvantage, and social and material adversity were measured through questionnaires and linked to register data on neighborhood disadvantage. Data was analyzed with longitudinal and cross-sectional multilevel models. Results showed that neighborhood disadvantage, social and material adversity and gender all contributed independently to overall levels of symptoms across the life course. Cross-sectional analyses also suggested that the impact of disadvantage differed between life course periods; neighborhood disadvantage was most important in young adulthood, and the relative importance of material versus social adversity increased as participants grew older. In summary, the study suggests that disadvantages from different contextual sources may affect functional somatic health across the life course, but also through life course specific patterns.

## Introduction

It is well-known that a plethora of burdening socioeconomic, psychosocial and material living conditions impacts on health. During the last decade or so conceptual models and empirical research has broadened the scope to include the surrounding environmental context, such as the neighborhood [1], [2]. However, research is severely lacking when it comes to incorporating a life course perspective on neighborhood disadvantage and health [3], [4], [5], particularly with regard to the fundamental question of when, during the life course, different forms of disadvantages impacts on health. The present study seeks to contribute to this inquiry.

As suggested by a collection of reviews and meta-analyses, disadvantaged neighborhood environments have the potential to impact on health across the life course; during infancy [6], [7], childhood and adolescence [8], [9], adulthood[1], [6], [10], [11], [12], [13], [14], as well as in old age [15]. Taken together, the neighborhood environment thus appears to be a context of importance for physical and mental health across the entire life course, and in most cases above and beyond the importance of individual circumstances. However, the substantial heterogeneity of studies, each one limited to a restricted period of the life course, hampers any inferences about developmental variability in effect; e.g. whether the neighborhood represents a more important context in early or later life. As Sampson et al. [16] point out, the neighborhood boundaries can represent a spatial restriction for at least young children's daily routines, which could mean that the neighborhood context is particularly relevant for the well-being of children compared to later in life [8]. But so far, this has not been studied.

When considering the neighborhood context as an environmental source of poor health, it is also important to consider the more immediate environmental contexts in which people live their lives. As an example, the household have been argued as a more important unit of analysis for variations in adult mortality than the broader contexts of census tract and municipality [17]. Like for neighborhood influences, disadvantages rooted in the proximal environment can affect health differentially across the life course. This is, for example, suggested by the family economic stress model by Conger & Conger [18], [19], which posits that material circumstances, while affecting parents directly, impact on adolescents more indirectly through interpersonal pathways of parental mental health, marital discord and in the end, parenting behavior.

It is thus plausible that different forms of disadvantage may have differential importance for health during specific periods of life. A challenge when formulating such environmental and developmental contrast is the choice of appropriate health outcome. For the present paper we have chosen to focus on functional somatic symptoms (FSS), bodily complaints that cannot be confidently attributed to organic pathology [20]. Functional somatic symptoms are common in both adults [21] and young people [22], display a measure of continuity over the life course [22], [23], and the number of FSS in adolescence has been shown to be prognostic of various mental disorders in adulthood [22], [24]. Regardless of whether the somatic complaints are functional or not, FSS have a strong influence on quality of life and health status [21] and are closely interrelated with anxiety and depressive symptoms [25]. As such, FSS may be an appropriate example of health that is relevant to study across the life course. Moreover, early studies indicate that not only personal living conditions, but also one's residential context may be relevant for FSS [26].

In this paper, we will approach the topic of health effects of disadvantage in a sample of adolescents growing up in a Northern Swedish municipality in the early 1980s, and who have been followed across young adulthood in mid-1980s and 1990s, up to mid-life in 2007. Sweden has a long-standing tradition of an ideological emphasis on equality and de-commodification [27], e.g. expressed in high unionization, public health policies explicitly focusing on reducing health inequalities, well-developed social security systems and universal health care. This legislative and ideological context would be expected to mitigate the risk of individuals exposed to severe disadvantage which may be more common in other parts of the world. Health inequalities in the Swedish context can therefore be expected to be of a moderate degree from a global perspective. However, Sweden as a welfare state has also undergone changes, with increased income inequalities since the mid-1990s [28] and an ideological shift towards a more neo-liberal agenda, which is also seen in changes to public health policies in the 2000s [27]. Accordingly, the study should be viewed within the context of three decades of what has been labeled a declining welfare state [27].

The general research question we pose is: do neighborhood and individual (socioeconomic, social and material) disadvantages relate to health across the life course? The specific aims are: 1) To examine whether disadvantages display an *overall* effect on self-reported FSS from adolescence to mid-adulthood; and 2) To investigate if disadvantages relate to FSS *at specific life course periods* (age 16, 21, 30 and 42 years).

## Methods

### Ethics statement

Ethical approval was granted by the Regional Ethical Review Board in Umeå. The retrieval and use of register data was also approved through a separate review of data safety and confidentiality by Statistics Sweden. Separate written informed consent was not requested by either committee, as the participants were regarded as giving written consent when completing the questionnaire at each data collection wave. All participants were clearly informed that participation in the study is voluntary and that they can decide to withdraw from participation at any time, without giving any explanation.

### Sample and general procedures

The initial setting of the study is the Northern Swedish municipality of Luleå, in which all participants were living in adolescence. The majority of the municipality's population reside in Luleå City, with about 45 000 inhabitants. Luleå City is an industrial town with an emphasis on metallurgic industries, but also with a university, and is comparable to Sweden as a whole with regard to e.g. labor market structure, housing, housing and socioeconomic status, but has a history of higher levels of unemployment in the 1980s [29].

The Northern Swedish Cohort (NSC) is based on all school-leavers of the 9^th^ grade, the final grade of the Swedish compulsory school system, in the municipality of Luleå, in the year 1981, when the majority of participants were 16 years old. The eligible sample includes all who attended school as well as those who should have finished school this year but who had quit prematurely (n = 11). Individuals who went to special schools due to severe learning disability, visual impairment or hearing impairment were excluded, as well as one individual who was in long-term coma. There were 1083 eligible individuals, 1080 of whom participated in 1981. Four follow-up data collections waves (1983, 1986, 1995, 2007) have since then been conducted, at participant age 18, 21, 30 and 42 years; see Hammarström and Janlert [30] for details of the NSC procedures. At the latest data collection in 2007, n = 1010 participated (94.3% of those 1071 individuals of the original sample still alive), 1001 of whom participated in the part of the study including retrieval of register data. The Northern Swedish Cohort is conducted at Umeå University. For privacy reasons, the dataset is not freely available, but researchers interested in collaboration should get into contact with the Principal Investigator, Anne Hammarström.

For the present paper, questionnaire data from the age 16, 21, 30 and 42 data collection waves were used. The questionnaires have had similar overall content across the ages, but have been generally expanded in the later data collections, and have also been revised to make them appropriate for the respective ages. In addition, neighborhood of residence for all participants at the four measure points was linked to register data from Statistics Sweden, covering all residents in the respective neighborhoods.

Due to missing data on particular variables, the analytical sample varies between n = 857 and n = 992 in different analyses, corresponding to 80.0–92.6% of the original cohort still alive (n = 1071) and 85.6–99.1% of those participating in adulthood (n = 1001).

### Neighborhood data procedures

Neighborhoods were demarcated according to the SAMS (Small-area market statistics) areas, a small-scale geographical division of Sweden by Statistics Sweden, with an average of about 1000 individuals living in each area. The areas are constructed as polygons with demarcations made at roads and similar physically visible borders, with the intention to group buildings of similar type and appearance. As such, the SAMS areas correspond closely to what is commonly perceived as the immediate neighborhood.

All SAMS areas in which at least one cohort member resided according to the Swedish population registry, on December 31^st^ in 1980, 1986, 1995 and 2007, respectively, were included as neighborhoods. During 1981, many participants moved from home in conjunction with leaving school, and therefore neighborhood of residence in 1980 was chosen for the 1981 measure point. Due to participants moving between the measure points, the number of neighborhoods increased from n = 72 in 1981, n = 215 in 1986, n = 333 in 1995 to n = 374 in 2007. The median number of residents per neighborhood varied between n = 979 (1981) to n = 1400 (2007).

For all residents living in any of the above specified neighborhoods at the specified time points, variables as close as possible to the measure points were retrieved from registers of Statistics Sweden. Occupational status was only available in 1980 and 1985 and used as proxy for the 1981 and 1986 measure points, while educational level was only available for 1995 and 2007. Furthermore, single parent status for the 1981 and 1986 measure points was based on variables from 1980 and 1985, respectively, and information on wealth tax for 2007 is based on data from 2006. For all other variables, information is from 1981, 1986, 1995 and 2007.

### Measures

All measures were operationalized at the ages 16, 21, 30 and 42 years, with the aim of yielding equivalent measures at the different ages. Neighborhood disadvantage was based on register data while information for the other measures came from the self-administered questionnaires the participants completed at each data collection wave.

#### Functional somatic symptoms (FSS)

Functional somatic symptoms was operationalized as the sum of 10 symptoms during the last 12 months [31]: *headache, migraine; other stomach ache*; *nausea; backache, hip pain, sciatica; fatigue; breathlessness; dizziness; overstrain* (all with three response options: no ( = 0); yes, light ( = 1); severe ( = 2)); *palpitations* (three response options never ( = 0); sometimes ( = 1); often/always ( = 2)); and *sleeping difficulties* (four response options with the two highest collapsed into a single category: never ( = 0); sometimes ( = 1); often or always ( = 2)).

Internal consistency was estimated at Cronbach alpha  =  0.70 (age 16 years), 0.70 (age 21), 0.74 (age 30) and 0.78 (age 42).

#### Neighborhood disadvantage (ND)

Neighborhood disadvantage was operationalized at each age as a combination of eight indicators, with the aim of broadly covering socioeconomic conditions in a consistent manner [31], [32]. The selection of indicators was guided by previous research [2], [33] and by the availability of indicators in the registers. See Table 1 for details of the operationalization and descriptive statistics.

**Table 1:** Operationalization of neighborhood disadvantage indicators at each age of participants (mean percentages across neighborhoods).

Indicator and operationalization	Age 16 (n = 72)	Age 21 (n = 215)	Age 30 (n = 333)	Age 42 (n = 374)
1) Low income: Percentage of individuals in a household *with an annual disposable household income per consumption unit* in the household ≤10^th^ percentile of the Swedish population the corresponding year	10.4	10.0	9.2	7.8
2) High income: Percentage of individuals living in a household *with an annual disposable household income* per *consumption unit* in the household ≥90^th^ percentile of the Swedish population the corresponding year (reverse coded)	9.9	9.9	10.8	12.1
3) Housing allowance: Percentage of individuals living in household receiving housing allowance	18.4	12.2	19.5	5.4
4) Wealth: Percentage of individuals paying any amount of wealth tax (reverse coded)	1.7	4.8	3.8	3.2
5) Non-employment: Percentage of adults (≥18yrs) whose main income is from *unemployment*, *early retirement*, or *sickness benefits* or *compensation*; not counting income from retirement or employment	7.4	5.9	11.8	6.7
6) Single parent: Percentage of individuals living in single-parent households with one or more children	10.9	20.0	7.7	7.8
7a) Low occupational status: Percentage of individuals living in household with unskilled manual worker (SEI: 11–12) as the highest occupational level.	16.1	20.2	—	—
7b) Low educational achievement: Percentage of individuals ≥25yrs with only primary education, including primary education <9 years, and primary education 9–10) years	—	—	25.8	15.1
8a) High occupational status: Percentage of individuals living in household with professionals or self-employed (SEI: 56–60) as the highest occupational level (reverse coded)	11.0	15.7	—	—
8b) High educational achievement: Percentage of individuals ≥25yrs with 2 or more years of tertiary education or PhD (reverse coded)	—	—	28.3	33.6

SEI =  Socioeconomic Classification scheme by Statistics Sweden [“Socioekonomisk indelning” in Swedish].

The indicators selected were percentages of neighborhood residents with 1) *Low income*, 2) *High income* (reverse coded), 3) *Housing allowance*, 4) *Wealth* (reverse coded), 5) *Non-employment*, 6) *Single-parent household*, 7a) *Low occupational status* (only available for 1981 and 1986), 7b) *Low educational attainment* (only available for 1995 and 2007), 8a) *High occupational status* (reverse coded; only available for 1981 and 1986), and 8b) *High educational achievement* (reverse coded; only available for 1995 and 2007).

Based on the eight indicators, neighborhood disadvantage scores were calculated as the mean of the Z-scores of the eight indicators, separately for each age.

Internal consistency (Cronbach's α) for the life-course specific scores was estimated at α = 0.89 (1981); α = 0.81 (1986); α = 0.86 (1995); and α = 0.89 (2007) at the neighborhood level, and α = 0.93 (1981); α = 0.88 (1986); α = 0.85 (1995); and α = 0.86 (2007) at the individual level.

#### Individual social and material adversity

Adversity was operationalized as the sum of different burdening life circumstances, selected from those available in the questionnaires at each age [34]. Separate measures were constructed for social adversity, comprising exposures which could involve threats to salient relationships, and material adversity, which consisted of exposures related to physical living conditions or the financial situation. All adversities were binary or dichotomized as close as possible to the 80^th^ percentile.

Social adversity comprised the following adversities: parental loss/separation, residential instability up to age 16, parental illness (at age 16); residential instability during the last three years, illness of a close one, death of a close one (at age 21); separation/divorce, illness of a close one, death of a close one, social isolation, low decision latitude, and exposure to threat/violence (at both age 30 and 42).

Material adversity consisted of the following adversities: poor material standard of living, residential crowding, parental unemployment (at age 16); low cash margin, low income, unemployment (at age 21); low cash margin, financial strain, unemployment, and spousal unemployment (at both age 30 and 42).

#### Individual socioeconomic disadvantage (SED)

Socioeconomic disadvantage was based on occupation and operationalized as manual worker ( = 1) and non-manual employee or self-employed ( = 0) according to the classification scheme of Statistics Sweden [35], [36]. At age 21, 30 and 42 the participant's own occupation was used, and at age 16 the occupation of the parents with both parents belonging to manual workers coded as 1, and at least one parent being non-manual employee or self-employed coded as 0. For parents (age 16) and participants (age 42) not working, the last held occupation was considered. Last held occupation was not recorded at age 21 and 30, so at these ages highest educational attainment was used as a proxy for individuals not in gainful employment, with university-preparatory high school or university coded as 0 and vocational high school or less coded as 1. For participants who only lived with one of the parents, only the occupation of the parent with whom they were living was considered.

### Data analysis

To examine whether the small drop-out was systematic, missingness (defined as missing data one or more of the variables included in the analyses) was regressed on each variable (neighborhood disadvantage, socioeconomic disadvantage, social adversity, material adversity, gender, and FSS at age 16, 21, 30 or 42), in separate logistic regression models. Neither variable was significantly related to missingness (p>.05) and as such no evidence for systematic drop-out was found. Therefore, complete case analysis was used in the main analyses.

Hierarchical linear regression models were used as the main statistical method, using the user-written *runmlwin* command in Stata to fit multilevel models in the MLwiN software package v.2.27 [37]. All but the binary variables were standardized before entered in the model. Starting with restricted iterative generalized least squares estimation, we applied a Markov Chain Monte Carlo (MCMC) estimation procedure, with a burn-in of 500, a chain length of 5,000 and a thinning interval of 1 [38]. Fixed effects are reported as regression coefficients with 95% credible intervals (CrI), which can be interpreted in a similar way to confidence intervals [38].

To address our first specific aim, a series of three-level longitudinal hierarchical linear regression models were run. In these analyses, the outcome FSS over the life course was regressed on time-varying covariates, with time (i.e. survey year at participant age 16, 21, 30 and 42) representing the level 1 unit, which was nested within individuals (level 2 unit), which were nested within neighborhoods (level 3 unit). First, an empty model was run without any predictors (Model 0). Subsequent models included different sets of predictors: time (Model 1), time and ND (Model 2), time and individual-level predictors (gender, socioeconomic disadvantage, social adversity, material adversity; Model 3), and in a final model, all predictors from the preceding models (Model 4).

To address our second aim, a series of cross-sectional two-level hierarchical linear regression analyses were fitted, in which individuals (level 1) were nested within neighborhoods (level 2) separately for each life course period (age 16, 21, 30 and 42). In these models, individual-level FSS at each age was regressed on predictors measured concurrently with the outcome. The following models were run at each age: empty model (Model 0), ND (Model 1), individual level predictors (Model 2) and a fully adjusted model with all predictors (Model 3). In addition, differential effects of disadvantage by life course period were tested by adding a Time × Predictor interaction terms for each predictor to a three level model as described above.

Exploratory analyses stratified for gender were done to explore whether the finding were valid for both women and men. As the analyses yielded estimates comparable to those in the total sample (results not shown), only results from the collapsed sample are reported in the results section.

## Results

### Bivariate analyses

See Table 2 for descriptive statistics and bivariate associations between individual-level predictors and concurrent FSS at age 16, 21, 30 and 42 years. Functional somatic symptoms decreased between age 16 to age 21, with a successive increase to age 30 and 42, and women reported more FSS than men at all life course periods. Socioeconomic disadvantage was related to higher FSS at all periods except at age 16, while both social and material adversity were positively associated to FSS at all surveys.

**Table 2:** Descriptive statistics of individual-level variables, and bivariate correlation (Pearson's r) with concurrent FSS at age 16, 21, 30 and 42.

Variable, estimate	Age 16 (n = 992)		Age 21 (n = 966)		Age 30 (n = 902)		Age 42 (n = 986)	
	Descriptive	r (p)	Descriptive	r (p)	Descriptive	r (p)	Descriptive	r (p)
FSS, M (SD)	3.35 (2.54)	−	2.82 (2.521	−	3.73 (2.93)	−	4.24 (3.31)	−
Gender, n (%) women	482 (48.15)	−0.13*	482 (48.15)	−0.14*	482 (48.15)	−0.11*	482 (48.15)	−0.15*
Socioeconomic disadvantage, n (%) manual workers	377 (38.04)	0.04	623 (63.06)	0.10*	425 (43.37)	0.12*	349 (34.87)	0.13*
Social adversity, M (SD)	0.73 (0.81)	0.17*	0.76 (0.85)	0.18*	0.97 (0.99)	0.22*	1.64 (1.26)	0.33*
Material adversity, M (SD)	0.49 (0.68)	0.09*	0.54 (0.68)	0.09*	0.77 (0.95)	0.23*	0.44 (0.75)	0.35*

FSS = Functional Somatic Symptoms.

*p<.05.

### Does disadvantage have an overall impact on FSS over the life course?

See table 3 for results from a longitudinal hierarchical regression of FSS across the life course periods (at age 16, 21, 30 and 42 years) regressed on neighborhood and individual disadvantage.

**Table 3:** Summary of three-level hierarchical linear regression with time nested within individuals, nested within neighborhoods: functional somatic symptoms regressed on neighborhood and individual disadvantage over the life course.

Estimates	Model 0	Model 1	Model 2	Model 3	Model 4
Cons	0.01 (−0.03–0.06)	−0.28 (−0.36–−0.20)	−0.18 (−0.27–−0.10)	0.20 (0.07–0.32)	0.25 (0.12–0.38)
Time (continuous)		0.11 (0.09–0.14)	0.08 (0.05–0.10)	0.05 (0.03–0.08)	0.04 (0.01–0.07)
**Area level fixed effects**					
ND			0.11 (0.07–0.15)		0.05 (0.01–0.09)
**Individual level fixed effects**					
Gender (ref: women, vs men)				−0.23 (−0.30–−0.17)	−0.24 (−0.30–−0.17)
SED (ref: manual, vs non-manual)				0.06 (−0.01–0.12)	0.04 (−0.02–0.11)
Social adversity				0.21 (0.18–0.24)	0.20 (0.17–0.23)
Material adversity				0.13 (0.10–0.16)	0.13 (0.10–0.16)
**Random effects**					
Area level variance	0.01	0.0	0.00	0.00	0.00
Individual level variance	0.40	0.39	0.38	0.29	0.30
Time level variance	0.60	0.59	0.58	0.59	0.58
Bayesian DIC	10124.70	10074.59	9984.14	9816.56	9762.96

Numbers are regression coefficients and 95% Credible Intervals if not otherwise noted.

ND = Neighborhood Disadvantage; SED = Socioeconomic Disadvantage; DIC = Deviance Information Criterion.

First, concerning random effects in the empty model (Model 0), 40% of the total variance in FSS over time was attributed to clustering by individuals, with a mere 1% to clustering by neighborhood.

Second, some patterns emerged in the fixed effects (Table 3, Model 1–4). As indicated by the time estimates, life course period displayed a modest influence on average levels of FSS, with an average 0.11 SD increase in FSS for each consecutive survey (Model 1). Neighborhood disadvantage also showed a positive relationship to FSS over the life course, with one SD increase in ND corresponding to 0.11 SD increase in FSS (Model 2). Although the ND regression coefficient was markedly attenuated by the addition of individual-level predictors (Model 4), it remained significant. Of the individual-level predictors, social and material adversity presented independent and robust associations to FSS, with more adversity corresponding to more FSS over the life course (Model 2 and 3). In contrast to the bivariate analysis, the estimate for socioeconomic disadvantage did not reach significance in any of the models. Women reported more FSS over the life course than did men, even accounting for disadvantages (Model 3–4).

### Does disadvantage impact on FSS at specific life course periods?

Results from cross-sectional analyses at specific life course period are shown in Table 4 (age 16 years), Table 5 (age 21), Table 6 (age 30) and Table 7 (age 42).

**Table 4:** Summary of two-level hierarchical linear regression with individuals nested within neighborhoods: functional somatic symptoms regressed on neighborhood and individual disadvantage at age 16 years.

Estimates	Model 0	Model 1	Model 2	Model 3
Cons	0.00 (−0.07–0.07)	0.00 (−0.07–0.08)	0.38 (0.17–0.58)	0.37 (0.17–0.58)
**Area level fixed effects**				
ND		−0.02 (−0.09–0.05)		−0.09 (−0.17–−0.02)
**Individual level fixed effects**				
Gender (ref: women, vs men)			−0.26 (−0.38–−0.13)	−0.26 (−0.39–−0.14)
SED (ref: manual, vs non-manual)			0.05 (−0.08–0.18)	0.09 (−0.05–0.22)
Social adversity			0.16 (0.10–0.22)	0.17 (0.11–0.24)
Material adversity			0.04 (−0.02–0.11)	0.06 (−0.00–0.12)
**Random effects**				
Area level variance	0.02	0.02	0.02	0.01
Individual level variance	0.99	0.99	0.94	0.94
VPC	0.02	0.02	0.02	0.01
Bayesian DIC	2815.22	2816.66	2761.12	2758.53

Numbers are regression coefficients and 95% Credible Intervals if not otherwise noted.

ND = Neighborhood Disadvantage; SED = Socioeconomic Disadvantage; VPC =  Variance Partition Coefficient; DIC = Deviance Information Criterion.

**Table 5:** Summary of two-level hierarchical linear regression with individuals nested within neighborhoods: functional somatic symptoms regressed on neighborhood and individual disadvantage at age 21 years.

Estimates	Model 0	Model 1	Model 2	Model 3
Cons	−0.00 (−0.07–0.06)	−0.00 (−0.07–0.06)	0.23 (0.02–0.45)	0.22 (0.02–0.43)
**Area level fixed effects**				
ND		0.10 (0.04–0.17)		0.06 (0.00–0.13)
**Individual level fixed effects**				
Gender (ref: women, vs men)			−0.25 (−0.37–−0.12)	−0.23 (−0.35–−0.11)
SED (ref: manual, vs non-manual)			0.23 (0.10–0.36)	0.20 (0.07–0.33)
Social adversity			0.16 (0.10–0.22)	0.16 (0.09–0.28)
Material adversity			0.09 (0.03–0.15)	0.09 (0.02–0.15)
**Random effects**				
Area level variance	0.00	0.00	0.00	0.00
Individual level variance	0.99	0.98	0.94	0.94
VPC	0.00	0.00	0.00	0.00
Bayesian DIC	2745.35	2710.31	2672.24	2645.21

Numbers are regression coefficients and 95% Credible Intervals if not otherwise noted.

ND = Neighborhood Disadvantage; SED = Socioeconomic Disadvantage; VPC =  Variance Partition Coefficient; DIC = Deviance Information Criterion.

**Table 6:** Summary of two-level hierarchical linear regression with individuals nested within neighborhoods: functional somatic symptoms regressed on neighborhood and individual disadvantage at age 30 years.

Estimates	Model 0	Model 1	Model 2	Model 3
Cons	−0.00 (−0.07–0.06)	−0.01 (−0.08–0.05)	0.22 (0.01–0.42)	0.29 (0.08–0.49)
**Area level fixed effects**				
ND		0.16 (0.09–0.23)		0.11 (0.04–0.18)
**Individual level fixed effects**				
Gender (ref: women, vs men)			−0.18 (−0.31–−0.05)	−0.22 (−0.34–−0.09)
SED (ref: manual, vs non-manual)			0.11 (−0.02–0.25)	0.06 (−0.07–0.20)
Social adversity			0.16 (0.10–0.23)	0.15 (0.08–0.22)
Material adversity			0.17 (0.10–0.24)	0.15 (0.08–0.22)
**Random effects**				
Area level variance	0.01	0.00	0.00	0.01
Individual level variance	0.99	0.96	0.91	0.89
VPC	0.01	0.00	0.01	0.01
Bayesian DIC	2563.46	2479.53	2460.50	2391.39

Numbers are regression coefficients and 95% Credible Intervals if not otherwise noted.

ND = Neighborhood Disadvantage; SED = Socioeconomic Disadvantage; VPC =  Variance Partition Coefficient; DIC = Deviance Information Criterion.

**Table 7:** Summary of two-level hierarchical linear regression with individuals nested within neighborhoods: functional somatic symptoms regressed on neighborhood and individual disadvantage at age 42 years.

Estimates	Model 0	Model 1	Model 2	Model 3
Cons	0.00 (−0.07–0.08)	−0.00 (−0.07–0.07)	0.33 (0.15–0.52)	0.36 (0.17–0.55)
**Area level fixed effects**				
ND		0.11 (0.04–0.18)		0.01 (−0.06–0.07)
**Individual level fixed effects**				
Gender (ref: women, vs men)			−0.23 (−0.34–−0.11)	−0.24 (−0.36–−0.13)
SED (ref: manual, vs non-manual)			0.04 (−0.09–0.16)	0.03 (−0.10–0.16)
Social adversity			0.23 (0.17–0.29)	0.24 (0.18–0.30)
Material adversity			0.25 (0.18–0.31)	0.23 (0.17–0.30)
**Random effects**				
Area level variance	0.07	0.03	0.02	0.01
Individual level variance	0.94	0.96	0.80	0.81
VPC	0.06	0.03	0.03	0.02
Bayesian DIC	2789.58	2708.28	2603.51	2534.13

Numbers are regression coefficients and 95% Credible Intervals if not otherwise noted.

ND = Neighborhood Disadvantage; SED = Socioeconomic Disadvantage; VPC =  Variance Partition Coefficient; DIC = Deviance Information Criterion.

Area of residence (Model 0) explained an insubstantial percentage of FSS variance at all ages except age 42, with the variance partition coefficient (VPC) estimated at 2% at age 16, <1% at age 21, 1% at age 30, and 6% at age 42.

The association between neighborhood disadvantage and FSS displayed a measure of variation across the life course periods (Model 1, Table 4–7; interaction term p<.001). In adolescence, ND was not related to FSS in the unadjusted model (Model 1), but showed a significant negative relationship with FSS, i.e. more disadvantage corresponding to lower FSS levels, after adjustment for individual-level predictors (Model 3). In contrast, in young adulthood (age 21 and 30), ND was significantly and positively related to FSS both before (Model 1) and after (Model 3) adjustment for individual-level predictors. In middle-age (age 42) ND was significant only in the unadjusted model (Model 1), with an estimate of comparable strength as at age 21 and 30, but attenuated below significance after inclusion of individual-level predictors (Model 3).

Although the impact of social adversity was numerically stronger than material adversity on overall FSS across the life course (Table 3), the cross-sectional analyses indicated a gradual shift of their relative importance for concurrent FSS as participants grew older. Starting at age 16 (Table 4), social adversity seemed to be of greater importance for concurrent FSS than was material adversity, with the latter estimate being non-significant (Model 2–3). In adulthood (age 21, 30 and 42), however, both social and material adversity displayed independent contributions to concurrent FSS, with the point estimate of material adversity matching that of social adversity at age 30 and 42. As a numerical illustration, the ratio of the social/material adversity point estimates decreased from 2.7 at age 16, to 1.8 at age 21, to 1.0 at age 30 and 42. Both social and material adversity displayed significant interaction with time (p <.001).

In contrast to the bivariate analyses (Table 2), socioeconomic disadvantage was only independently related to FSS at age 21 (Model 3, Table 5), with a just significant interaction with time (p = .031). Women reported more FSS at all ages even when neighborhood and individual disadvantage had been taken into account (Model 3), with the largest numerical difference present at age 42 but with non-significant interaction with time (p = .142).

## Discussion

To the best of our knowledge, this study is the first of its kind examining how different types of disadvantages relate to health at different periods of the life course. We found that neighborhood disadvantage, social and material adversity and gender independently contributed to overall levels of functional somatic symptoms over the life course. Moreover, we found that the relative importance of different kinds of disadvantage for concurrent symptoms was not necessarily constant over the life course; neighborhood disadvantage was a more prominent determinant of symptoms in young adulthood than in mid-adulthood, and even displayed a negative relationship to symptoms in adolescence. Social adversity displayed a numerically stronger association than material adversity to symptoms in adolescence, with the relative importance of material adversity growing with age.

Our findings suggest that neighborhood circumstances are relevant for mental health across the life course, and thus substantially extend early reports showing that FSS can relate to residential environments in the form of urban/rural contexts [26]. Nevertheless, symptoms were only marginally clustered by neighborhoods at most specific ages, indicating that the neighborhood may not be an efficient target for intervention [39]. A possible explanation to the generally low level of clustering is the comparatively low levels of residential segregation in Sweden, and particularly in the more Northern rural areas of Swedish. In the same vein, the comparatively high clustering in mid-adulthood could be indicative of the increasing socioeconomic inequalities in Sweden during the last decade [28], or alternatively explained by different needs, vulnerabilities and susceptibilities to environmental influences linked to the stages of the life course.

An independent contribution of neighborhood disadvantage was found only in young adulthood (age 21 and 30). It is possible that this is explained by young adulthood representing a particularly extrovert period in life after detachment from one's family of origin in late adolescence, as is the norm in Sweden, but before one's own family formation and parenthood, when the home environment again may become increasingly important. The surprising negative association in adolescence could, speculatively, be explained by protective parenting behavior limiting youths' access to the neighborhood environment, elicited by a hostile neighborhood environment. The finding could also reflect the hypothesized equalizing effect of peer groups in adolescence [40]. A study in Canadian children [41] also reported worse mental health in children of disadvantaged families in advantaged neighborhoods, and framed the findings within models of relative deprivation and competition for limited resources [42]. While Canada has some similarities to Sweden when it comes to historically well-developed but declining welfare systems [27], that particular study focused on urban areas with a minimum core population of 100.000. As neighborhood disadvantage in our study context would not be expected to correspond to concentrated poverty, or to other instances of limited material resources, the relative deprivation model may be more appropriate here.

The results concerning social and material adversity tentatively suggest that in adolescence, interpersonal burdens could be more important for health complaints, but that material circumstances become increasingly important after one's transition into adulthood. This finding is in accordance with Conger & Conger's economic stress model [18], [19], which illustrates how the developmental impact of material circumstances on adolescents is mediated by interpersonal pathways, such as marital discord and parenting behaviors. Adults, on the other hand, have financial responsibility, and are thus expected to be more directly affected by material conditions. Alternative explanation could be that the reduced social security systems of Sweden make personal material conditions more important determinants for well-being, or changed susceptibilities to environmental influences along the life course.

The unadjusted analyses (Table 2) and the main analyses (Table 3–7) together suggest that socioeconomic disadvantage does have an influence on health complaints, but that it is mediated by social and/or material adversity. This conclusion is similar to what we have reported previously for cumulative biological risk outcomes [34].

### Methodological considerations

The general strengths of the present study includes a sample representative of similar age cohorts of Sweden, a long follow-up time with several survey waves, and with data from multiple sources. Attrition rate was also very low, which is protective against selection bias, and we were unable to demonstrate systematic patterns in the missing data.

The sample has been found to be comparable to similar age cohorts of Sweden on a number of background variables [29], promoting external validity. However, the overall historical context, e.g. changes in cultural patterns, macroeconomics and demographics, and the individual developmental contexts, e.g. life trajectories of the individual and her or his living environments, were impossible to empirically disentangle in this study. For example, the eroding welfare system in Sweden during the 2000s is a contextual transformation which should be expected to influence how disadvantage impacts on health over the life course.

The definition of geographical boundaries is a crucial issue in area effects on health research [17], [43], [44], and the use of administrative boundaries such as in this study may not necessarily represent valid boundaries for clustering of health. As such, the weak clustering up to mid-age could potentially be a result of inappropriate boundaries, or a sign of temporal variability of the validity or boundaries.

Both the outcome and the neighborhood disadvantage measures were operationalized identically at all surveys and also have a similar content to other functional somatic symptoms questionnaires [20] and neighborhood disadvantage operationalizations [2], [33]. In contrast, the set of individual-level adversities differed by age, which was necessary in order to make the adversities age-relevant, but which also may limit the comparison of the adversity estimates between specific life course periods. For a detailed methodological discussion about the adversity measures, see Gustafsson et al. [34].

As highlighted by discussions from a counterfactual framework [43], [45], causal inference in area effects on health research also presents a number of problems, since the individuals living in different neighborhoods may not be exchangeable with each other, e.g. due to residential selection into disadvantaged neighborhoods by poor health or other personal conditions. Although we have recently demonstrated residential selection effects in adulthood by social circumstances in adolescence [31], the combination of repeated cross-sectional and longitudinal analyses in the present report should mitigate the problem of residential selection due to poor health or other factors.

The geographical dispersion has increased over the study years, with a consequential reduction in neighborhood cluster sizes. Simulation studies have found little evidence for small cluster sizes (including singletons) biasing at least fixed estimates as long as the number of level-2 units are large (>50), but have found biased estimates particularly for level-2 random estimates with few level 2 units [46], [47]. Others have found that the area level variance may be overestimated with small cluster sizes [48], which may be particularly relevant for the interpretation of the cross-sectional analyses at age 42 in the present study. Despite these potential problems, authors unanimously warn against reverting to single-level analyses such as OLS regression [47], [48].

### Conclusions

The present prospective study, conducted in the context of a declining Nordic welfare state, shows that disadvantage rooted in both the neighborhood and immediate environments have an overall importance for levels of self-reported functional somatic symptoms across the life course. The results also suggest that there is a measure of variability in the health impact of different forms of disadvantage across the life course. Neighborhood disadvantage showed the greatest negative influence in young adulthood, and material adversity became increasingly important as one grew older, and the gender gap in symptoms was widest in mid-life. Future research should explore developmental variability in how disadvantage from different sources impact on health across the life course.
